# Analytical and clinical challenges in a patient with concurrent type 1 diabetes, subcutaneous insulin resistance and insulin autoimmune syndrome

**DOI:** 10.1530/EDM-13-0086

**Published:** 2014-04-01

**Authors:** N Jassam, N Amin, P Holland, R K Semple, D J Halsall, G Wark, J H Barth

**Affiliations:** Harrogate District HospitalHarrogate, HG2 7SXUK; 1 Leeds Children's Hospital NHS TrustLeedsUK; 2 Wellcome TrustCambridge University HospitalCambridgeUK; 3 Clinical Biochemistry DepartmentAddenbrooke's HospitalCambridgeUK; 4 SAS Peptides Hormone SectionRoyal Surrey County HospitalSurreyUK; 5 Blood Sciences DepartmentLeeds Teaching Hospitals NHS TrustLeedsUK

## Abstract

A lean 15-year-old girl was diagnosed with type 1 diabetes based on symptomatic hyperglycaemia and positive anti-islet cell antibodies. Glycaemia was initially stabilised on twice-daily mixed insulin. After 11 months from the time of diagnosis, she complained of hyperglycaemia and ketosis alternating with hypoglycaemia. This progressively worsened until prolonged hospital admission was required for treatment of refractory hypoglycaemia. A high titre of anti-insulin antibodies was detected associated with a very low recovery of immunoreactive (free) insulin from plasma after precipitation with polyethylene glycol, suggesting the presence of insulin in bound complexes. Insulin autoimmune syndrome was diagnosed and metabolic fluctuations were initially managed supportively. However, due to poor glucose control, immunosuppressive therapy was initiated first with steroids and plasmapheresis and later with anti-CD20 antibody therapy (Rituximab). This treatment was associated with a gradual disappearance of anti-insulin antibodies and her underlying type 1 diabetes has subsequently been successfully managed with an insulin pump.

**Learning points:**

- Anti-insulin antibodies may result in low levels of free insulin.
- Polyclonal anti-insulin antibodies can interfere with the pharmacological action of administered insulin, resulting in hypoglycaemia and insulin resistance, due to varying affinities and capacities.
- In this patient, rituximab administration was associated with a gradual disappearance of anti-insulin antibodies.
- It is hypothesised that this patient had subcutaneous insulin resistance (SIR) caused by insulin capture at the tissue level, either by antibodies or by sequestration.
- A prolonged tissue resistance protocol may be more appropriate in patients with immune-mediated SIR syndrome.

## Background

Insulin autoimmune syndrome (IAS or Hirata's disease) is a common cause of hypoglycaemia and mild insulin resistance that has most frequently been described in Japan, but is very rare within the Caucasian population (1). In this condition, circulating insulin autoantibodies are generated against endogenous insulin in patients who have not been exposed to exogenous insulin (2).

Among diabetic patients treated with recombinant human insulin, antibodies created against exogenous insulin are also a common phenomenon and hypoglycaemia has been seen in cases where insulin antibodies are present in the circulation (3). Hypoglycaemia in insulin-treated patients may occur because of the release of the hormone from the circulating insulin–antibody complex, but in general, these antibodies rarely affect the course of the disease, the daily insulin requirements or the glycaemic control (4). However, there have been a few cases worldwide where patients with type 1 diabetes treated with recombinant human insulin have developed a high titre of circulating insulin antibodies. The presence of these antibodies has led to unstable glycaemic control, resulting in a severe form of insulin resistance (5) (6). Previous treatment has been with double filtration plasmapheresis followed by prednisolone and/or mycophenolate mofetil (5) (6).

Insulin resistance associated with s.c. insulin administration is another uncommon condition that complicates T1DM management (7). This condition is characterised by decreased sensitivity to s.c., but not i.v. insulin. The pathophysiology of subcutaneous insulin resistance (SIR) is not well understood. Other than immune-mediated insulin resistance, mechanisms such as increased enzymatic activity leading to rapid insulin degradation at the injection site, poor insulin diffusion or insulin sequestration in the adipose tissue have been proposed but not sufficiently documented (8) (9).

In this paper, we report a patient with type 1 diabetes with a form of immune-mediated insulin resistance, in which the insulin resistance has been developed as a result of the presence of circulating insulin antibodies, with possible tissue resistance to subcutaneous insulin. We have described the clinical course of this patient, with use of surrogate markers to monitor disease activity and treatment modalities used to maintain normoglycaemia.

## Case presentation

A lean 15-year-old white Caucasian female was diagnosed with type 1 diabetes. At the time of diagnosis, the patient presented with polyuria, polydipsia and a random glucose of 20 mmol/l. She tested positive for anti-gliadin antibodies and islet cell antibodies. She had no significant illness prior to this and there was no family history of diabetes or any other autoimmune diseases. Examination found no features of insulin resistance. She had good glycaemic control during the first 6 months following diagnosis. Her plasma HbA1c was maintained at 58 mmol/mol with s.c. Mixtard 30, in a dose of 32 units twice daily, which was increased to four times a day within two months of diagnosis. She had never received animal-derived insulin preparation.

After 8 months from the time of diagnosis, the patient reported cyclical swings in her insulin requirements according to her menstrual cycle. Her insulin requirements in the first 2 weeks of the cycle were around 60 units/day. This was followed by a week where the insulin requirement rose to 90–120 units/day, and within 4 months her insulin requirements were 280 units/day, with little effect on blood glucose levels. This period was usually followed by a week when she required no insulin to be administered (average glucose of 3.2 mmol/l).

After 2 months, the cyclical swings of insulin became worse. Her insulin requirements continued to rise up to 300 U/day. These periods were usually preceded by intervals of hyperglycaemia and ketosis that resulted in repeated admission to the intensive care unit, where the patient was managed with i.v. insulin. Furthermore, the period when insulin was not required became shorter and was followed by long periods of hypoglycaemia. The hypoglycaemic episodes were very severe, with un-recordable glucose concentrations, and the patient required continuous infusions with 15–20% dextrose at an infusion rate of 200 ml/h for up to 2 weeks. Any interruption in i.v. infusion of dextrose led to the immediate recurrence of hypoglycaemia. These episodes became recurrent and nocturnal. Surreptitious insulin self-administration or insulin administration by proxy was considered but no evidence was found.

## Investigation

In view of the cyclical swings in insulin requirements, a number of laboratory tests were performed. Biochemical tests for luteinising and follicle hormones, thyroid hormones, cortisol and growth hormone were all within the normal range. During episodes of severe hypoglycaemia, when i.v. insulin administration had been stopped, blood insulin concentrations were markedly elevated at 10 750 pmol/l, with appropriate C-peptide levels of <94 pmol/l. Insulin and C-peptide levels vary greatly depending on glucose levels, but in patients with no anti-insulin antibodies the ratio is typically maintained at 1:5–1:10 (insulin:C-peptide). In patients with anti-insulin antibodies, the half-life for insulin becomes much longer, making the results more difficult to interpret.

In the insulin immunoassay, the high titre of the circulating anti-insulin antibodies is considered to be the interfering substance (10), hence insulin measurement alone becomes unreliable. Instead, insulin recovery as a percentage of free insulin (before polyethylene glycol (PEG) 6000 precipitation) to total insulin after PEG precipitation is required to give an accurate functioning insulin level. On the patient's admission to our centre, free insulin recovery was remarkably low (it varied from 9 to 17%) and the patient was strongly positive for anti-insulin antibodies.

**Figure 1:**
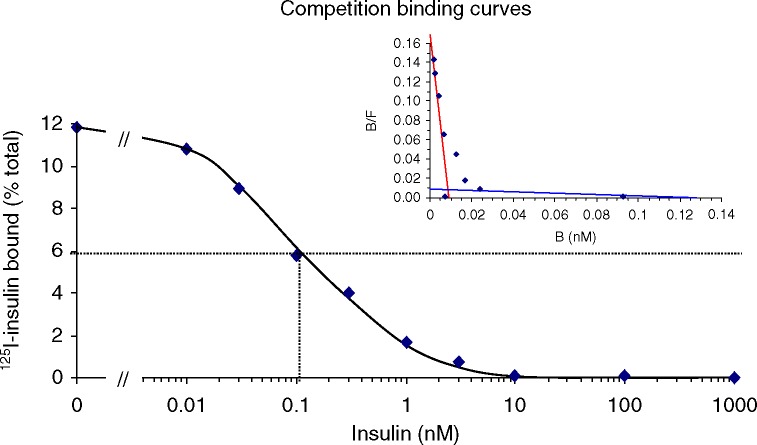
Scatchard analysis of the insulin autoantibodies in this patient using ^125^I human insulin. B:F, bound:free ratio. The specific binding is expressed as a percentage of the total insulin. The Scatchard plots of insulin–antibody binding data showed a bimodal distribution, suggesting that two classes of antibodies exist. Two orders of sites could be inferred from this plot – a high-affinity population of binding sites with a *K*_a_ of ∼2×10^10^/M and a low capacity and a lower affinity population with a *K*_a_ of ∼6×10^7^/M and a higher capacity.

Figure 1 illustrates the Scatchard analysis of the anti-insulin antibodies in this patient. The results revealed the presence of two populations of polyclonal IgG antibodies. One population had high affinity (*K*_a_ ≈2×10^10^/M) with low capacity (0.009 nm in the assay, ∼0.8 nm antibody in the serum) and the other had low affinity (*K*_a_ ≈6×10^7^/M) but higher capacity (0.13 nm in the assay, ∼12 nm antibody in the serum). However, half maximal inhibition of ^125^I-insulin binding occurs with ∼0.1 nm insulin, suggesting that most of the binding in this assay is due to high-affinity binding sites. The shape of the curve is relatively shallow with complete displacement occurring over more than a 100-fold concentration of unlabelled insulin, suggesting that insulin is binding to polyclonal antibodies. This analysis showed that the characteristics of circulatory antibodies are similar to those associated with insulin autoimmune syndrome (IAS). These studies were conducted after trials of plasmapheresis, immunoglobulins, prednisolone and methotrexate to improve glycaemic control. Rituximab had not yet been used.

**Figure 2:**
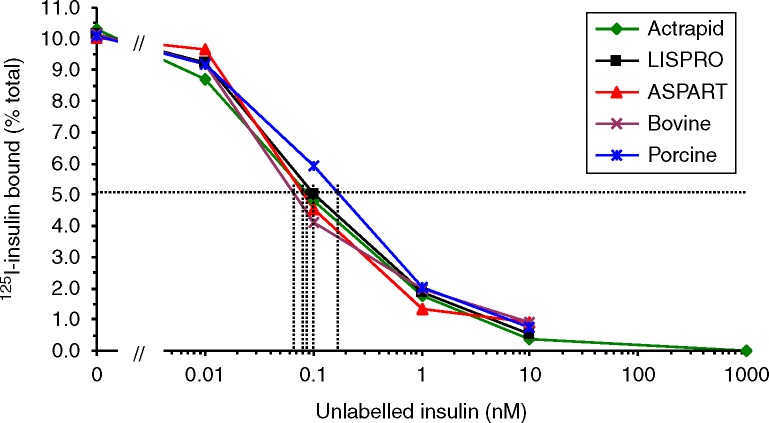
The affinity of binding various insulin preparations to insulin autoantibodies positive sample. Displacement of ^125^I binding to the patient serum by various types of insulin. The specific binding (i.e. NSB in the presence of 10 μM insulin has been subtracted) is expressed as a percentage of the total insulin. Binding of human ^125^I-insulin is decreased with increasing concentrations of all the insulin preparations tested, with a very similar half maximal inhibition of ^125^I-insulin binding for the human analogues and bovine insulin (0.07–0.1 nM).

Figure 2 illustrates the affinity of these antibodies in binding to different insulin preparations. There was no demonstrable difference in the ability of the tested insulin preparations to compete with the labelled hormones for antibody-binding sites, indicating that our patient had a similar affinity for all the tested insulin preparations.

**Figure 3:**
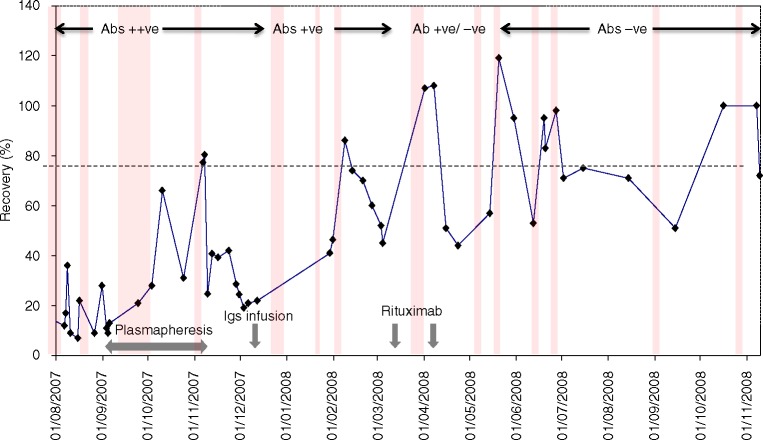
Insulin recovery in relation to different treatment regimes and the concentration of insulin antibodies over the first 14 months of hospitalisation. The broken horizontal line indicates a normal recovery. The vertical lines represent the hypoglycaemic episodes. The width of each line is an estimate for the duration of hypoglycaemic period. The widest line represents a 2-week period.

Figure 3 shows insulin recovery, episodes of hypoglycaemia and concentration of insulin antibodies in response to different treatment modalities over the first 14 months of hospitalisation. The different treatment modalities are outlined in the section below.

**Figure 4:**
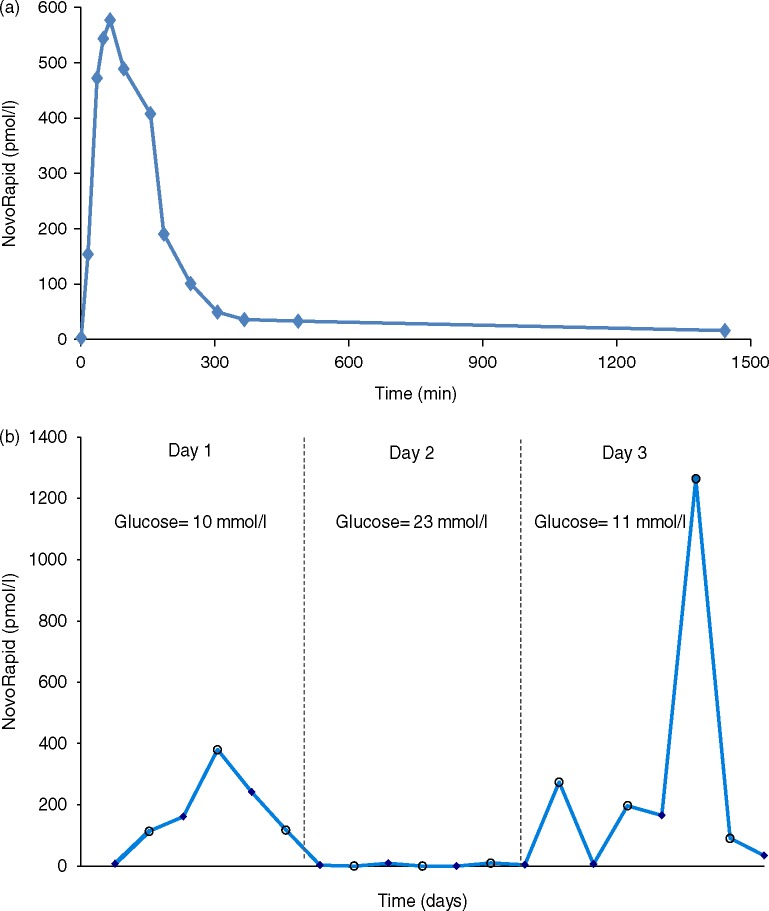
(a and b) The insulin analogue, NovoRapid, concentration in the patient's circulation during 24 h tissue resistance test (a) and during a prolonged test duration (b). The solid circle in (b) represents the insulin level before insulin injection. The open circle represents the insulin level after insulin injection. NovoRapid of 20 units was injected in the 24 h test and 10 U were injected after each meal in the 3 days duration test. NovoRapid of 10 U is equivalent to 300 pmol/l. The glucose levels documented are the average glucose concentrations on each of the 3 days.

Subsequent to all immunosuppressant therapies, and 9–12 months after use of rituximab, tissue-resistant tests were performed (Fig. 4a and b). The first test was conducted over 24 h (Fig. 4a) and the second over 36 h (Fig. 4b). The glucose values in Fig. 4b represent the progressive increase in glucose concentration despite insulin administration, indicating that insulin may have been trapped in the subcutaneous tissue and released later in the circulation. This is outlined in further detail in the discussion section.

## Treatment

Owing to the presence of circulating antibodies, it was elected to treat the patient as having IAS. Hence, she had three sessions of double-filtration plasmapheresis and was started on 60 mg prednisolone. During this time, she had three episodes of severe hypoglycaemia. Prednisolone treatment worsened glycaemic control, hence the dose was gradually reduced to 5 mg daily.

Although plasmapheresis successfully removed insulin antibodies from the circulation (as indicated by insulin recovery >70% and negative anti-insulin antibodies), this effect was short lived and antibodies reappeared in the circulation a few hours after the plasmapheresis session or after s.c. insulin administration.

Immunoglobulin infusion and 20 mg methotrexate weekly were also tried and were found to be unsuccessful in reducing the severity of the nocturnal hypoglycaemia or degree of insulin resistance. In view of the failure of all the treatment modalities, a decision was made to give the patient rituximab.

The patient was given the first dose of rituximab and started on methylprednisolone. Subsequent monitoring showed that within 6 months the circulating antibodies disappeared. A >70% insulin recovery with a negative insulin antibody titre was sustained for the second year of hospitalisation (Fig. 3). Although the nocturnal hypoglycaemia became less severe and shorter in interval, the patient experienced five episodes over the subsequent 14 months and insulin resistance to subcutaneous insulin did not change.

During this time in an attempt to improve the patient's glycaemic control, i.v. Actrapid and the insulin analogue, glargine, administrated subcutaneously, were used to manage the patient. Although, the anti-insulin antibodies were diminished from the circulation, this treatment modality resulted in worsening glycaemic control and precipitated hypoglycaemia lasting for 3 days.

Over the subsequent years, episodes of hypoglycaemia diminished and she could be managed purely with continuous subcutaneous insulin infusion (CSII) via an insulin pump. In total, the patient was hospitalised for 28 months before becoming stable enough for discharge.

## Outcome and follow-up

After 60 months from the time of use of rituximab and methylprednisolone, the patient has become stable and uses a CSII via an insulin pump to manage her type 1 diabetes. She has had no further periods of hospitalisation for over 5 years and her insulin requirement is ∼38 units/day.

## Discussion

This is a complex case of immune-mediated insulin resistance presented with unusually severe hypoglycaemia. This young lady with type 1 diabetes initially presented with circulating anti-insulin antibodies. Anti-insulin antibodies to human insulin preparation in diabetic children are not an uncommon phenomena and the presence of these antibodies in the patient's circulation is associated with an increase in the frequency of hypoglycaemia (8) (11). The anti-insulin antibody characteristics are one of the causative factors of hypoglycaemic episodes. The characteristics of anti-insulin antibodies have been thoroughly studied in IAS. It is evident that high-affinity insulin antibodies frequently induce insulin resistance, whereas the presence of low-affinity antibodies induce hypoglycaemia due to the dissociation of the high amount of insulin from the insulin–antibody complex (12) (13). However, in diabetes, only a few studies have focused on the characteristics of these antibodies (6). In this case, anti-insulin antibodies interfered with the pharmacological action of administrated insulin resulting in hypoglycaemia and insulin resistance. The autoimmune hypothesis for the origin of the anti-insulin antibodies in this patient was tested by characterising those antibodies and studying their association constant and binding capacity. The characterisation of anti-insulin antibodies in this patient confirmed the existence of polyclonal insulin antibodies. This finding is consistent with the concept that the antibodies to exogenous insulin are generally considered to be polyclonal and consist of various components with different affinities and capacities (14). Furthermore, the values of association affinity constant and capacity reported in this case were similar to those reported in association with IAS in non-diabetics (15). The diagnosis of IAS as the sole cause of the patient symptoms was refuted after she still had an ongoing insulin requirement despite lack of insulin antibodies in the circulation.

Indeed, the clinical history always suggested dual pathology as the patient maintained a high sensitivity to i.v. insulin but not to s.c. insulin. This phenomenon is consistent with SIR, which complicated the case history of this patient.

In 1979, Paulsen *et al*. defined SIR according to three criteria:

- resistance to the action of subcutaneous insulin but maintaining sensitivity to i.v. insulin;
- lack of increase in the circulatory insulin after subcutaneous insulin and
- increased insulin degrading activity in the subcutaneous tissue (16).

While the last criterion represents a small percentage of cases of published SIR, the first criterion has been met in our case. Fulfilment of the second criteria is less clear in this case. Our data from the first tissue resistance test (Fig. 4A), which was performed over 24 h, revealed a normal response with an insulin peak at around 40 min. This was a similar finding to that in normal diabetics as presented by Hedman *et al*. (18). When this test has been repeated but over a period of three days (Fig. 4B), the patient showed no rise in circulating insulin but a concomitant rise in glucose. On the third day and while the same dose of insulin has been given subcutaneously, a massive peak of insulin, inappropriate to the administered dose, appeared in the circulation, with a concurrent fall in glucose concentration. Although only based on the clinical history of one patient we may conclude that in immune-mediated SIR and where there is a gradual build up to subcutaneous insulin, an insulin tissue resistance protocol performed over few days is more appropriate than the 24 h test.

Given our finding, we hypothesise that the mechanism causing insulin resistance starts when the subcutaneously administrated insulin is captured in the tissues. Few case reports have linked SIR to rapid insulin degradation at the injection site (17). This mechanism is very unlikely given the evidence that supports the subsequent release of insulin into the circulation.

We postulate two mechanisms that may explain insulin resistance and hypoglycaemia in this patient. The first is consistent with the presence of anti-insulin antibodies or receptors, at the tissue level. We hypothesise that tissue antibodies were formed through an immune reaction against exogenous insulin. The gradual rise in requirement of insulin over time followed by a sudden appearance of insulin in the circulation is consistent with an immune-mediated process, where a gradual filling up to antibody-binding sites leads to a saturation point at which the bound insulin and insulin antibodies are freed into the circulation. However, the mechanism that governs the insulin antibodies and insulin release process is not understood. The administered insulin is subsequently captured by circulating insulin antibodies. The buffering effect of the circulating insulin antibodies worsens insulin resistance and contributes to the increase in insulin requirements. The fasting state encourages the release of a large amount of the bound insulin and results in the nocturnal hypoglycaemia. What is not known is whether the circulating antibodies were dissociated from the tissue antibodies or they were produced separately, although insulin recovery studies during plasmapheresis showed the immediate return of insulin antibodies after s.c. insulin administration. However, autoimmune disorders sometimes coexist in one patient and this case may represent the coexistence of dual pathologies.

This hypothesis is further supported by the reduction in length and severity of hypoglycaemia when the patient was anti-insulin antibody negative. Indeed, at this phase, the glycaemic control was adequately managed with i.v. insulin on its own. Hypoglycaemia usually appeared a few days or weeks after s.c. insulin administration, indicating that the autoimmune reaction to subcutaneous insulin was resolving.

The second postulated mechanism is consistent with insulin sequestration in the derma or adipose tissue and subsequent release into the circulation and the presence of circulating insulin antibodies can be a concurrent finding that complicated the tissue resistance picture. However, evidence from the clinical presentation and patient response to immunosuppression therapy is in favour of the earlier hypothesis. Although the second mechanism appears less likely, it cannot be eliminated.

The mechanism of the immunogenicity of the exogenous human anti-insulin antibody is still unknown. Several factors are considered to influence the production of insulin antibodies, such as genetic factors, mode of insulin administration, degree of purity and species of insulin. It also appears that different insulin analogues induced different immune reactions in this patient. That was evident by the severity and the length of hypoglycaemic episode after administration of long-acting insulin in this patient.

The use of differential insulin assays with different specificities for different types of exogenous insulin was an important factor that helped explaining the kinetics of administered insulin analogues. Using these assays, we first demonstrated that high levels of insulin analogue, NovoRapid, were detectable during episodes of hypoglycaemia, although this was not administered on the same day. This observation, together with those from the prolonged tissue resistant test, was important in understanding the mechanism of the disease, informing management and education of the patient and family. The abnormal response of the prolonged tissue resistant tests is consistent with a gradual build-up of tissue resistance. Evidence from early clinical presentation showed a gradual increase in insulin requirement and the concomitant rise in glucose concentrations over periods of days preceded a period of severe hypoglycaemia. This indicates that the more the site of injection was exposed to subcutaneous insulin, the more severe the picture of insulin resistance was. Over the 28 months of hospitalisation, it becomes apparent that tissue resistance to subcutaneous insulin worsened after a period of regular s.c. injection and regressed following a period of discontinuation. Indeed, the 24 h tissue resistance test was preceded by a 2 weeks period of discontinuation of s.c. injections, which may explain the normal response seen from this test. The tissue resistance tests and the differential insulin assay results elucidated the skin involvement in the disease mechanism.

It is known that genetic predisposition is important in the pathogenesis of IAS. This has been suggested by an apparent HLA association with IAS cases in which typing has been performed. It has been found that 96% of IAS Japanese patients possessed the DR4, Cw4 and B15 antigens and almost all of them carried HLA-DRB1*0406, HLA-DQA1*0301/HLA-DQB1*0302 (1). Although our patient was positive for DR4, DNA sequencing indicated that the HLA-DRB1*0406 allele was not present. In the Caucasian population, of the seven cases studied, four possessed DR4 and carried HLA-DRB1*0401 and three had non-DR4 phenotypes either with HLA-DRB1*0101, HLA-DRB1*1501 or HLA-DRB1*0701. Our patient's HLA typing would seem to support the hypothesis of IAS in that she is positive for DR4 (HLA-DRB1*0401) and HLA-DRB1*0101 (19).

Various treatment modalities for SIR such as immunosuppression, plasmapheresis, protease inhibitors, insulin with heparin, peritoneal insulin administration, insulin analogue, inhaled insulin and β-cell transplantation have been proposed but were inconsistently effective, which may reflect several pathophysiologies for this condition.

Treating IAS patients with steroids, immunoglobulin infusions and plasmapheresis (6) is well established. In our patient, treatment with steroids partially lowered the insulin antibodies titre but worsened glycaemic control. Treatment with plasmapheresis failed to sustain a response due to recurrence of insulin autoantibodies after s.c. insulin administration. I.v. insulin administration improved the glycaemic control but was complicated by central line infection episodes. The immunoglobulin infusion had no impact on the course of the disease. Therefore, a potent immunosuppressant drug rituximab was administrated. Rituximab is a B-cell-depleting agent, with potential risk and side effects. This treatment was associated with a gradual disappearance of anti-insulin antibodies. Indeed, it took a few months before the insulin antibodies were diminished and 14 months after rituximab administration before the hypoglycaemic spells faded and the insulin resistance resolved. After 3 years, good glycaemic control was maintained.

It is not clear if the hyperglycaemic episodes were related to the SIRS or from IAS. Both IAS and SIR can be associated with hypo- and hyperglycaemia, and we cannot be certain to which the hyperglycaemia was attributable. Episodes of hyperglycaemia frequently preceded the episodes of hypoglycaemia, even after eradication of circulating anti-insulin antibodies, suggesting that hyperglycaemia may have been attributable to SIR as well as IAS.

In conclusion, this was a challenging clinical scenario where immune-mediated tissue resistance and circulatory insulin antibodies may underline the pathophysiology in this patient. It was the first time that insulin recovery studies were used routinely as a surrogate marker of disease activity. The laboratory had a major role in this case and the use of the differential insulin assays has shed the light on the kinetics of insulin resistance in this patient. Furthermore, it is obvious from this case that a short tissue resistant test as proposed by Pualsen *et al*. (16) is not sufficient to exclude SIR. A prolonged tissue resistance protocol may be more appropriate in patients with immune-mediated SIR.

## Patient's perspective

What I thought was a simple chronic condition of type 1 diabetes suddenly changed my way of life completely. Within months of my diagnosis with diabetes I was regularly admitted into my local hospital with fluctuating blood sugar levels. I was having seizures due to low blood sugar levels followed by episodes of ketosis with high blood sugar levels. Being diagnosed with diabetes was one thing but less than a year later I had a unique condition which did not appear to have a name nor could anyone answer any of our questions and that was the difficult part. Everything appeared to be a risk and a guessing game but I was fortunate to have Dr P Holland as my consultant, a remarkable dedicated and caring consultant who was always open and honest with my family and me along the way which made the journey that little bit easier. I put my life in his hands and trusted him completely. I did feel like I was in a bubble for the duration of my hospital stay but my condition is continuing to improve and I am thoroughly enjoying getting a part of my life back.

## Patient consent

Consent has been obtained from the patient.

## Author contribution statement

N Jassam provided biochemistry advice and helped in writing the article; N Amin contributed to the writing of the article and was the, clinician involved in care of patient. P Holland is the named physician of patient. R K Semple provided advice about the investigation, management of the patient and helped in the writing of the article. D J Halsall provided advice and development of insulin assays. G Wark contributed to sample analysis and data processing. J H Barth provided biochemistry advice.

## References

[1] Uchigata Y & Hirata Y . 1999 Insulin autoimmune syndrome (IAS, Hirata disease). Annales de Médecine Interne. 155: 245–253 10445096

[2] Uchigata Y , Eguchi Y , Takayama-Hasumi S & Omori Y . 1994 Clinical characteristics of 197 patients with insulin autoimmune syndrome in Japanese. Diabetes Research and Clinical Practice. 22: 89–94 10.1016/0168-8227(94)90040-X 8200300

[3] Van Haeften TW . 1989 Clinical significance of insulin antibodies in insulin-treated diabetic patients. Diabetes Care. 12: 641–648 10.2337/diacare.12.9.641 2676431

[4] Wredling R , Lins PE & Adamson U . 1990 Prevalence of antiinsulin antibodies and its relation to severe hypoglycaemia in insulin-treated diabetic patients. Scandinavian Journal of Clinical and Laboratory Investigation. 50: 551–557 10.3109/00365519009089170 2237267

[5] Segal T , Webb EA , Viner R , Pusey C , Wild G & Allgrove J . 2008 Severe insulin resistance secondary to insulin antibodies: successful treatment with the immunosuppressant MMF. Pediatric Diabetes. 9: 250–254 10.1111/j.1399-5448.2008.00408.x 18547238

[6] Koyama R , Nakanishi K , Kato M , Yamashita S , Kuwahara H & Katori H . 2005 Hypoglycemia and hyperglycemia due to insulin antibodies against therapeutic human insulin: treatment with double filtration plasmapheresis and prednisolone. American Journal of the Medical Sciences. 329: 259–264 10.1097/00000441-200505000-00007 15894868

[7] Soudan B , Girardot C , Fermon C , Verlet E , Pattou F & Vantyghem MC . 2003 Extreme subcutaneous insulin resistance: a misunderstood syndrome. Diabetes & Metabolism. 29: 539–546 10.1016/S1262-3636(07)70069-1 14631332

[8] Lahtela JT , Knip M , Paul R , Antonen J & Salmi J . 1997 Severe antibody-mediated human insulin resistance: successful treatment with the insulin analog Lispro. A case report. Diabetes Care. 20: 71–73 10.2337/diacare.20.1.71 9028697

[9] Gillard P , Ling Z , Lannoo M , Maes B , Maleux G , Pipeleers D , Keymeulen B & Mathieu C . 2004 β-Cell transplantation restores metabolic control and quality of life in a patient with subcutaneous insulin resistance. Diabetes Care. 27: 2243–2244 10.2337/diacare.27.9.2243 15333492

[10] Casesnovaes A , Mauri M , Dominguez JR , Alfayate R & Pico AM . 1998 Influence of antiinsulin antibodies on insulin immunoassay in the autoimmune insulin syndrome. Annals of Clinical Biochemistry. 35: 768–774 10.1177/000456329803500610 9838991

[11] Seewi O , Jaeger C , Bretzel RG & Schonau E . 2008 Insulin binding to antibodies is a risk factor for inexplicable severe hypoglycaemia in children with type I diabetes mellitus. Experimental and Clinical Endocrinology & Diabetes. 116: 1–5 10.1055/s-2007-1004565 18484562

[12] Dozio N , Scavini M , Beretta A , Sarugeri E , Sartori S , Belloni C , Dosio F , Savi A , Fazio F , Sodoyez JC & Pozza G . 1998 Imaging of the buffering effect of insulin antibodies in the autoimmune hypoglycaemic syndrome. Journal of Clinical Endocrinology and Metabolism. 83: 643–648 10.1210/jcem.83.2.4599 9467587

[13] Kure M , Katsura Y , Kosano H , Noritake M , Watanabe T , Iwaki Y , Nishigori H & Matsuoka T . 2005 A trial to assess the amount of insulin antibodies in diabetic patients by surface plasmon resonance. Internal Medicine. 44: 100–106 10.2169/internalmedicine.44.100 15750268

[14] Kumar D . 1986 Insulin antibodies: an analysis of immunoglobulin G sub-classes. Diabetes. 35: 189A

[15] Goldman J , Baldwin D , Rubenstein AH , Klink DD , Blackard WG , Fisher LK , Roe TF & Schnure JJ . 1979 Characterisation of circulating insulin and proinsulin binding antibodies in autoimmune hypoglycaemia. Journal of Clinical Investigation. 63: 1050–1059 10.1172/JCI109374 447827 PMC372048

[16] Paulsen EP , Courtney JW III & Duckworth WC . 1979 Insulin resistance caused by massive degradation of subcutaneous insulin. Diabetes. 28: 640–645 10.2337/diab.28.7.640 109340

[17] Chalmers T . 1987 Subcutaneous insulin resistance syndrome. New England Journal of Medicine. 316: 49–51 10.1056/NEJM198701013160112 3537795

[18] Hedman CA , Lindström T & Arnqvist HJ . 2001 Direct comparison of insulin Lispro and Aspart shows small differences in plasma insulin profiles after subcutaneous injection in type 1 diabetes. Diabetes Care. 24: 1120–1121 10.2337/diacare.24.6.1120 11375381

[19] Cavaco B , Uchigata Y , Porto T , Amparo-Santos M , Sobrinho L & Leite V . 2001 Hypoglycaemia due to insulin autoimmune syndrome: report of two cases with characterisation of HLA alleles and insulin autoantibodies. European Journal of Endocrinology. 145: 311–316 10.1530/eje.0.1450311 11517012

